# Understanding human perception by human-made illusions

**DOI:** 10.3389/fnhum.2014.00566

**Published:** 2014-07-31

**Authors:** Claus-Christian Carbon

**Affiliations:** ^1^Department of General Psychology and Methodology, University of BambergBamberg, Germany; ^2^Bamberg Graduate School of Affective and Cognitive Sciences (BaGrACS)Bamberg, Germany

**Keywords:** optical illusion, delusion, deception, reality, perception, representation, validity, truth

## Abstract

It may be fun to perceive illusions, but the understanding of how they work is even more stimulating and sustainable: They can tell us where the limits and capacity of our perceptual apparatus are found—they can specify how the constraints of perception are set. Furthermore, they let us analyze the cognitive sub-processes underlying our perception. Illusions in a scientific context are not mainly created to reveal the failures of our perception or the dysfunctions of our apparatus, but instead point to the specific power of human perception. The main task of human perception is to amplify and strengthen sensory inputs to be able to perceive, orientate and act very quickly, specifically and efficiently. The present paper strengthens this line of argument, strongly put forth by perceptual pioneer Richard L. Gregory (e.g., Gregory, 2009), by discussing specific visual illusions and how they can help us to understand the magic of perception.

## About the veridicality of perception

### The relationship between reality and object

Sensory perception is often the most striking proof of something factual—when we perceive something, we interpret it and take it as “objective”, “real”. Most obviously, you can experience this with eyewitness testimonies: If an eyewitness has “seen it with the naked eye”, judges, jury members and attendees take the reports of these *percepts* not only as strong evidence, but usually as fact—despite the active and biasing processes on basis of perception and memory. Indeed, it seems that there is no better, no more “proof” of something being factual knowledge than having perceived it. The assumed link between perception and physical reality is particularly strong for the visual sense—in fact, we scrutinize it only when sight conditions have been unfortunate, when people have bad vision or when we know that the eyewitness was under stress or was lacking in cognitive faculties. When people need even more proof of reality than via the naked eye, they intuitively try to touch the to-be-analyzed entity (if at all possible) in order to investigate it haptically. Feeling something by touch seems to be the ultimate perceptual experience in order for humans to speak of physical proof (Carbon and Jakesch, 2013).

We can analyze the quality of our perceptual experiences by standard methodological criteria. By doing so we can regularly find out that our perception is indeed mostly very reliable and also objective (Gregory and Gombrich, 1973)—but only if we employ standard definitions of “objective” as being consensual among different beholders. Still, even by meeting these methodological criteria, we cannot give something in evidence about physical reality. It seems that knowledge about the physical properties of objects cannot be gained by perception, so perception is neither “veridical” nor “valid” in the strict sense of the words—the properties of the “thing in itself” remain indeterminate in any empirical sense (Kant, 1787/1998). We “reliably” and “objectively” might perceive the sun going up in the morning and down in the evening; the physical relations are definitely different, as we have known at least since Nicolaus Copernicus’s proposed heliocentricism—it might also be common sense that the Earth is a spheroid for most people, still the majority of people have neither perceived the Earth as spherical nor represented it like that; one reason for this is that in everyday life contexts the illusion of a plane works perfectly well to guide us in the planning and execution of our actions (Carbon, 2010b).

### Limitations of the possibility of objective perception

The limitations of perception are even more far reaching: our perception is not only limited when we do not have access to the thing in itself, it is very practically limited to the quality of processing and the general specifications of our perceptual system. For instance, our acoustic sense can only register and process a very narrow band of frequencies ranging from about 16 Hz–20 kHz as a young adult—this band gets narrower and narrower with increasing age. Typically, infrasonic and ultrasonic bands are just not perceivable despite being essential for other species such as elephants and bats, respectively. The perception of the environment and, consequently, the perception and representation of the world as such, is different for these species—what would be the favorite music of an elephant, which preference would a bat indicate if “honestly asked”? What does infrasonic acoustics sound and feel like? Note: infrasonic frequencies can also be perceived by humans; not acoustically in a strict sense but via vibrations—still, the resulting experiences are very different (cf. Nagel, 1974). To make such information accessible we need transformation techniques; for instance, a Geiger-Müller tube for making ionizing radiation perceivable as we have not developed any sensory system for detecting and feeling this band of extremely high frequency electromagnetic radiation.

But even if we have access to given information from the environmental world, it would be an illusion to think of “objective perception” of it—differences in perception across different individuals seem to be obvious: this is one reason for different persons having different tastes, but it is even more extreme: even within a lifetime of one person, the perceptual qualities and quantities which we can process change. Elderly people, for instance, often have yellowish corneas yielding biased color perception reducing the ability to detect and differentiate bluish color spectra. So even objectivity of perceptions in the sense of consensual experience is hardly achievable, even within one species, even within one individual—just think of fashion phenomena (Carbon, 2011a), of changes in taste (Martindale, 1990) or the so-called cycle of preferences (Carbon, 2010a)! Clearly, so-called objective perception is impossible, it is an illusion.

### Illusory construction of the world

**Figure 1:**
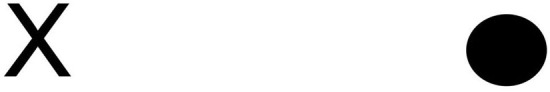
**Demonstration of the blind spot, the area on the retina where visual information cannot be processed due to a lack of photoreceptors**. The demonstration works as follows: Fixate at a distance of approx. 40 cm the X on the left side with your right eye while having closed your left eye—now move your head slightly in a horizontal way from left to right and backwards till the black disc on the right side seems to vanish.

The problem with the idea of veridical perception of the world is further intensified when taking additional perceptual phenomena, which demonstrate highly constructive qualities of our perceptual system, into account. A very prominent example of this kind is the perceptual effect which arises when any visual information which we want to process falls on the area of the retina where the so-called blind spot is located (see Figure 1).

Interestingly, visual information that is mapped on the blind spot is not just dropped—this would be the easiest solution for the visual apparatus. It is also not rigidly interpolated, for instance, by just doubling neighbor information, but intelligently complemented by analysing the meaning and Gestalt of the context. If we, for example, are exposed to a couple of lines, the perceptual system would complement the physically non-existing information of the blind spot by a best guess heuristic how the lines are interconnected in each case, mostly yielding a very close approximation to “reality” as it uses most probable solutions. Finally, we experience clear visual information, seemingly in the same quality as the one which mirrors physical perception—in the end, the “physical perception” and the “constructed perception”, are of the same quality, also because the “physical perception” is neither a depiction of physical reality, but is also constructed by top-down processes based on best guess heuristic as a kind of hypothesis testing or problem solving (Gregory, 1970).

Beside this prominent example which has become common knowledge up to now, a series of further phenomena exist where we can speak of full perceptual constructions of the world outside without any direct link to the physical realities. A very intriguing example of this kind will be described in more detail in the following: When we make fast eye movements (so-called saccades) our perceptual system is suppressed, with the result that we are functionally blind during such saccades. Actually, we do not perceive these blind moments of life although they are highly frequent and relatively long as such—actually, Rayner et al. estimated that typical fixations last about 200–250 ms and saccades last about 20–40 ms (Rayner et al., 2001), so about 10% of our time when we are awake is susceptible to such suppression effects. In accordance with other filling-in phenomena, missing data is filled up with the most plausible information: Such a process needs hypotheses about what is going on in the current situation and how the situation will evolve (Gregory, 1970, 1990). If the hypotheses are misleading because the underlying mental model of the situation and its further genesis is incorrect, we face an essential problem: what we then perceive (or fail to perceive) is incompatible with the current situation, and so will mislead our upcoming action. In most extreme cases, this could lead to fatal decisions: for instance: if the model does not construct a specific interfering object in our movement axis, we might miss information essential to changing our current trajectory resulting in a collision course. In such a constellation, we would be totally startled by the crash, as we would not have perceived the target object at all—this is not about missing an object but about entirely overlooking it due to a non-existing trace of perception.

Despite the knowledge about these characteristics of the visual system, we might doubt such processes as the mechanisms are working to so great an extent in most everyday life situations that it provides the perfect illusion of continuous, correct and super-detailed visual input. We can, however, illustrate this mechanism very easily by just observing our eye movements in a mirror: when executing fast eye movements, we cannot observe them by directly inspecting our face in the mirror—we can only perceive our fixations and the slow movements of the eyes. If we, however, film the same scene with a video camera, the whole procedure looks totally different: Now we clearly also see the fast movements; so we can directly experience the specific operation of the visual system in this respect by comparing the same scene captured by two differently working visual systems: our own, very cognitively operating, visual system and the rigidly filming video system which just catches the scene frame by frame without further processing, interpreting and tuning it.^1^ We call this moment of temporary functional blindness phenomenon “saccade blindness” or “saccade suppression”, which again illustrates the illusionary aspects of human perception “saccadic suppression”, Bridgeman et al., 1975; “tactile suppression”, Ziat et al., 2010). We can utilize this phenomena for testing interesting hypotheses on the mental representation of the visual environment: if we change details of a visual display during such functional blind phases of saccadic movements, people usually do not become aware of such changes, even if very important details, e.g., the expression of the mouth, are changed (Bohrn et al., 2010).

### Illusions by top-down-processes

**Figure 2:**
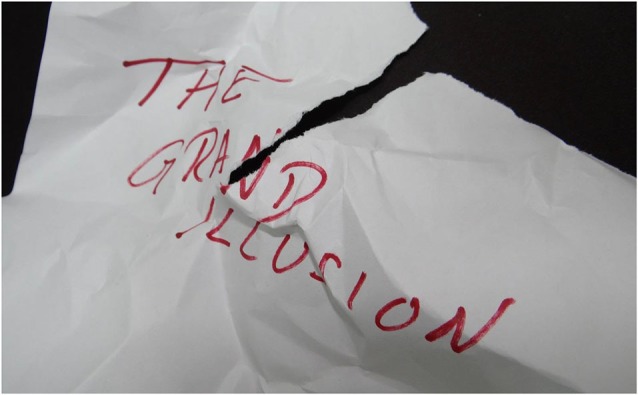
**Demonstration of top-down processing when reading the statement “The Grand Illussion” under highly challenging conditions (at least challenging for automatic character recognition)**.

Gregory proposed that perception shows the quality of hypothesis testing and that illusions make us clear how these hypotheses are formulated and on which data they are based (Gregory, 1970). One of the key assumptions for hypothesis testing is that perception is a constructive process depending on top-down processing. Such top-down processes can be guided through knowledge gained over the years, but perception can also be guided by pre-formed capabilities of binding and interpreting specific forms as certain Gestalts. The strong reliance of perception on top-down processing is the essential key for assuring reliable perceptual abilities in a world full of ambiguity and incompleteness. If we read a text from an old facsimile where some of the letters have vanished or bleached out over the years, where coffee stains have covered partial information and where decay processes have turned the originally white paper into a yellowish crumbly substance, we might be very successful in reading the fragments of the text, because our perceptual system interpolates and (re-)constructs (see Figure 2). If we know or understand the general meaning of the target text, we will even read over some passages that do not exist at all: we fill the gaps through our knowledge—we change the meaning towards what we expect.

**Figure 3:**
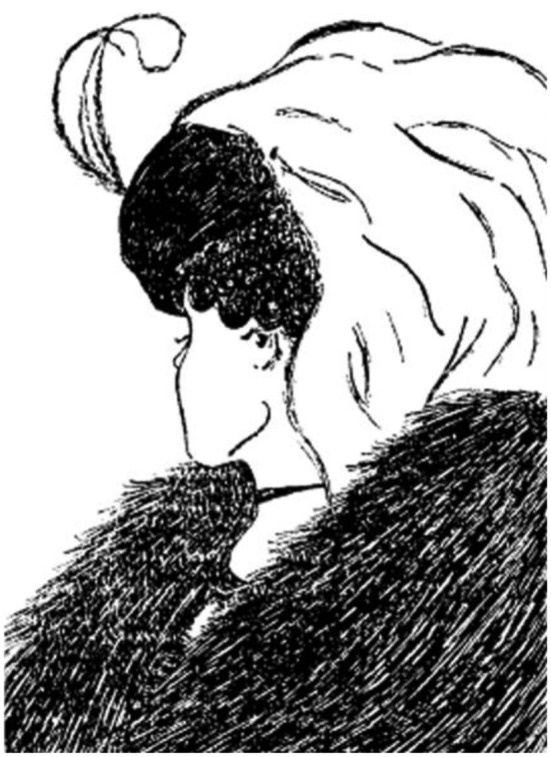
**The young-old-woman illusion (also known as the My Wife and My Mother-In-Law illusion) already popular in Germany in the 19th century when having been frequently depicted on postcards**. Boring (1930) was the first who presented this illusion in a scientific context (image on the right) calling it a “new” illusion (concretely, “a new ambiguous figure”) although it was very probably taken from an already displayed image of the 19th century within an *A and P Condensed Milk* advertisement (Lingelbach, 2014).

A famous example which is often cited and shown in this realm is the so-called man-rat-illusion where an ambiguous sketch drawing is presented whose content is not clearly decipherable, but switches from showing a man to showing a rat—another popular example of this kind is the bistable picture where the interpretation flips from an old woman to a young woman an v.v. (see Figure 3)—most people interpret this example as a fascinating illusion demonstrating humans’ capability of switching from one meaning to another, but the example also demonstrates an even more intriguing process: what we will perceive at first glance is mainly guided through the specific activation of our semantic network. If we have been exposed to a picture of a man before, or if we think of a man or have heard the word “man”, the chance is strongly increased that our perceptual system interprets the ambiguous pattern towards a depiction of a man—if the prior experiences were more associated with a rat, a mouse or another animal of such a kind, we will, in contrast, tend to interpret the ambiguous pattern more as a rat.

So, we can literally say that we perceive what we know—if we have no prior knowledge of certain things we can even overlook important details in a pattern because we have no strong association with something meaningful. The intimate processing between sensory inputs and our semantic networks enables us to recognize familiar objects within a few milliseconds, even if they show the complexity of human faces (Locher et al., 1993; Willis and Todorov, 2006; Carbon, 2011b).

Top-down processes are powerful in schematizing and easing-up perceptual processes in the sense of compressing the “big data” of the sensory inputs towards tiny data packages with pre-categorized labels on such schematized “icons” (Carbon, 2008). Top-down processes, however, are also susceptible to characteristic fallacies or illusions due to their guided, model-based nature: When we have only a brief time slot for a snapshot of a complex scene, the scene is (if we have associations with the general meaning of the inspected scene at all) so simplified that specific details get lost in favor of the processing and interpretation of the general meaning of the whole scene.

Biederman (1981) impressively demonstrated this by exposing participants to a sketch drawing of a typical street scene where typical objects are placed in a prototypical setting, with the exception that a visible hydrant in the foreground was not positioned on the pavement *besides* a car but unusually directly *on* the car. When people were exposed to such a scene for only 150 ms, followed by a scrambled backward mask, they “re-arranged” the setting by top-down processes based on their knowledge of hydrants and their typical positions on pavements. In this specific case, people have indeed been deceived, because they report a scene which was in accordance with their knowledge but not with the assessment of the presented scene—but for everyday actions this seems unproblematic. Although you might indeed lose the link to the fine-detailed structure of a specific entity when strongly relying on top-down processes, such an endeavor works quite brilliantly in most cases as it is a best guess estimation or approximation—it works particularly well when we are running out of resources, e.g., when we are in a specific mode of being pressed for time and/or you are engaged in a series of other cognitive processes. Actually, such a mode is the standard mode in everyday life. However, even if we had the time and no other processes needed to be executed, we would not be able to adequately process the big data of the sensory input.

The whole idea of this top-down processing with schematized perception stems from F. C. Bartlett’s pioneering series of experiments in a variety of domains (Bartlett, 1932). Bartlett already showed that we do not read the full information from a visual display or a narrative, but that we rely on schemata reflecting the essence of things, stories, and situations being strongly shaped by prior knowledge and its specific activation (see for a critical reflection of Bartlett’s method Carbon and Albrecht, 2012).

## Perception as a grand illusion

### Reconstructing human psychological reality

There is clearly an enormous gap between the big data provided by the external world and our strictly limited capacity to process them. The gap widens even further when taking into account that we not only have to process the data but ultimately have to make clear sense of the core of the given situation. The goal is to make one (and only one) decision based on the unambiguous interpretation of this situation in order to execute an appropriate action. This very teleological way of processing needs inhibitory capabilities for competing interpretations to strictly favor one single interpretation which enables fast action without quarrelling about alternatives. In order to realize such a clear interpretation of a situation, we need a mental model of the external world which is very clear and without ambiguities and indeterminacies. Ideally, such a model is a kind of caricature of physical reality: If there is an object to be quickly detected, the figure-ground contrast, e.g., should be intensified. If we need to identify the borders of an object under unfavorable viewing conditions, it is helpful to enhance the transitions from one border to another, for instance. If we want to easily diagnose the ripeness of a fruit desired for eating, it is most helpful when color saturation is amplified for familiar kinds of fruits. Our perceptual system has exactly such capabilities of intensifying, enhancing and amplifying—the result is the generation of schematic, prototypical, sketch-like perceptions and representations. Any metaphor for perception as a kind of tool which makes photos is fully misleading because perception is much more than blueprinting: it is a cognitive process aiming at reconstructing any scene at its core.

**Figure 4:**
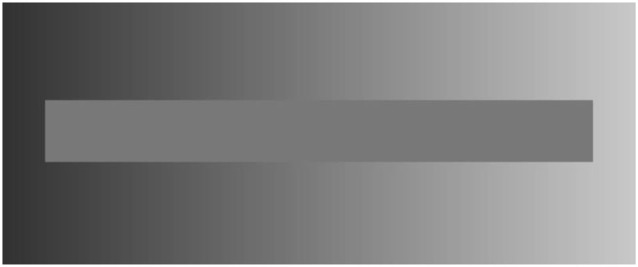
**Demonstration of the simultaneous contrast, an optical illusion already described as phenomenon 200 years ago by Johan Wolfgang von Goethe and provided in high quality and with an intense effect by McCourt (1982): the inner horizontal bar is** ***physically* filled with the same gray value all over, nevertheless, the periphery with its continuous change of gray from darker to lighter values from left to right induce the** ***perception* of a reverse continuous change of gray values**. The first one who showed the effect in a staircase of grades of gray was probably Ewald Hering (see Hering, 1907; pp. I. Teil, XII. Kap. Tafel II), who also proposed the theory of opponent color processing.

All these “intelligent perceptual processes” can most easily be demonstrated by perceptual illusions: For instance, when we look at the inner horizontal bar of Figure 4, we observe a continuous shift from light to dark gray and from left to right, although there is no physical change in the gray value—in fact only one gray value is used for creating this region. The illusion is induced by the distribution of the peripheral gray values which indeed show a continuous shift of gray levels, although in a reverse direction. The phenomenon of simultaneous contrast helps us to make the contrast clearer; helping us to identify figure-ground relations more easily, more quickly and more securely.

**Figure 5:**
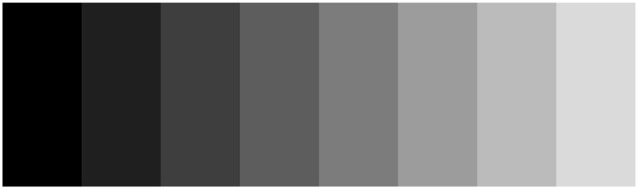
**Chevreul-Mach bands.** Demonstration of contrast exaggeration by lateral inhibition: although every bar is filled with one solid level of gray, we perceive narrow bands at the edges with increased contrast which does not reflect the physical reality of solid gray bars.

A similar principle of intensifying given physical relations by the perceptual system is now known as the Chevreul-Mach bands (see Figure 5), independently introduced by chemist Michel Eugène Chevreul (see Chevreul, 1839) and by physicist and philosopher Ernst Waldfried Josef Wenzel Mach (Mach, 1865). Via the process of lateral inhibition, luminance changes from one bar to another are exaggerated, specifically at the edges of the bars. This helps to differentiate between the different areas and to trigger edge-detection of the bars.

### Constructing human psychological reality

**Figure 6:**
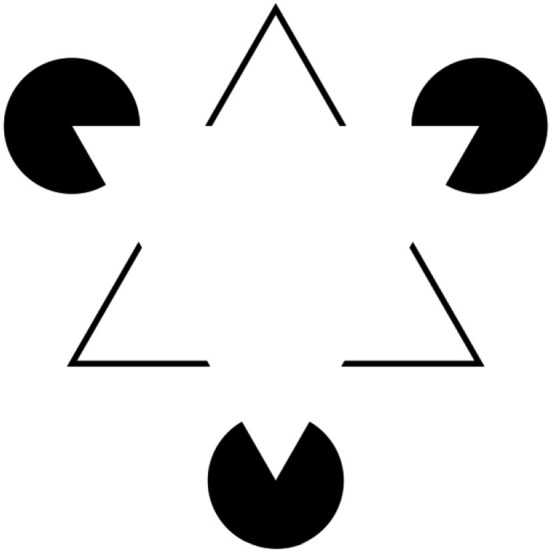
**Demonstration of illusory contours which create the clear perception of Gestalts**. The so-called Kanizsa triangle named after Gaetano Kanizsa (see Kanizsa, 1955), a very famous example of the long tradition of such figures displayed over centuries in architecture, fashion and ornamentation. We not only perceive two triangles, but even interpret the whole configuration as one with clear depth, with the solid white “triangle” in the foreground of another “triangle” which stands bottom up.

This reconstructive capability is impressive and helps us to get rid of ambiguous or indeterminate percepts. However, the power of perception is even more intriguing when we look at a related phenomenon. When we analyze perceptual illusions where entities or relations are not only enhanced in their recognizability but even entirely constructed without a physical correspondence, then we can quite rightly speak of the “active construction” of human psychological reality. A very prominent example is the Kanizsa triangle (Figure 6) where we clearly perceive illusory contours and related Gestalts—actually, none of them exists at all in a physical sense. The illusion is so strong that we have the feeling of being able to grasp even the whole configuration.

To detect and recognize such Gestalts is very important for us. Fortunately, we are not only equipped with a cognitive mechanism helping us to perceive such Gestalts, but we also feel rewarded when having recognized them as Gestalts despite indeterminate patterns (Muth et al., 2013): in the moment of the insight for a Gestalt the now determinate pattern gains liking (the so-called “Aesthetic-Aha-effect”, Muth and Carbon, 2013). The detection and recognition process adds affective value to the pattern which leads to the activation of even more cognitive energy to deal with it as it now means something to us.

## Conclusions

Perceptual illusions can be seen, interpreted and used in two very different aspects: on the one hand, and this is the common property assigned to illusions, they are used to entertain people. They are a part of our everyday culture, they can kill time. On the other hand, they are often the starting point for creating insights. And insights, especially if they are based on personal experiences through elaborative processes actively, are perfect pre-conditions to increase understanding and to improve and optimize mental models (Carbon, 2010b). We can even combine both aspects to create an attractive learning context: by drawing people’s attention via arousing and playful illusions, we generate attraction towards the phenomena underlying the illusions. If people get really interested, they will also invest sufficient time and cognitive energy to be able to solve an illusion or to get an idea of how the illusion works. If they arrive at a higher state of insight, they will benefit from understanding what kind of perceptual mechanism is underlying the phenomenon.

We can of course interpret perceptual illusions as malfunctions indicating the typical limits of our perceptual or cognitive system—this is probably the standard perspective on the whole area of illusions. In this view, our systems are fallible, slow, malfunctioning, and imperfect. We can, however, also interpret illusory perceptions as a sign of our incredible, highly complex and efficient capabilities of transforming sensory inputs into understanding and interpreting the current situation in a very fast way in order to generate adequate and goal-leading actions in good time (see Gregory, 2009)—this view is not yet the standard one to be found in beginners’ text books and typical descriptions or non-scientific papers on illusions. By taking into account how perfectly we act in most everyday situations, we can experience the high “intelligence” of the perceptual system quite easily and intuitively. We might not own the most perfect system when we aim to reproduce the very details of a scene, but we can assess the core meaning of a complex scene.

Typical perceptual processes work so brilliantly that we can mostly act appropriately, and, very important for a biological system, we can act in response to the sensory inputs very fast—this has to be challenged by any technical, man-made system, and will always be the most important benchmark for artificial perceptual systems. Following the research and engineering program of *bionics* (Xie, 2012),where systems and processes of nature are transferred to technical products, we might be well-advised to orient our developments in the field of perception to the characteristic processing of biological perceptual systems, and their typical behavior when perceptual illusions are encountered.

## Conflict of interest statement

The author declares that the research was conducted in the absence of any commercial or financial relationships that could be construed as a potential conflict of interest.

## Footnotes

^1^There is an interesting update in technology for demonstrating this effect putting forward by one of the reviewers. If you use the 2nd camera of your smartphone (the one for shooting “selfies”) or your notebook camera and you look at your depicted eyes very closely, then the delay of building up the film sequence is seemingly a bit longer than the saccadic suppression yielding the interesting effect of perceiving your own eye movements directly. Note: I have tried it out and it worked, by the way best when using older models which might take longer for building up the images. You will perceive your eye movements particular clearly when executing relatively large saccades, e.g., from the left periphery to the right and back.
